# Application of Theiler’s murine encephalomyelitis virus in treatment of multiple sclerosis

**DOI:** 10.3389/fmicb.2024.1415365

**Published:** 2024-06-26

**Authors:** Lin Li, Rui Zhou, Lin Sun

**Affiliations:** ^1^Third Hospital of Shanxi Medical University, Shanxi Medical University,Shanxi Bethune Hospital, Shanxi Academy of Medical Sciences, Tongji Shanxi Hospital, Taiyuan, China; ^2^School of Basic Medical Sciences, Shanxi Medical University, Taiyuan, China; ^3^First Hospital of Shanxi Medical University, Shanxi Medical University, Taiyuan, China

**Keywords:** TMEV, MS, gut microbiota, immune modulators, S1PR regulators, nervous repair agents

## Abstract

Theiler’s murine encephalomyelitis virus (TMEV) infected mice have been often used as an animal model for Multiple sclerosis (MS) due to their similar pathology in the central nervous system (CNS). So far, there has been no effective treatment or medicine to cure MS completely. The drugs used in the clinic can only reduce the symptoms of MS, delay its recurrence, and increase the interval between relapses. MS can be caused by many factors, and clinically MS drugs are used to treat MS regardless of what factors are caused rather than MS caused by a specific factor. This can lead to inappropriate medicine, which may be one of the reasons why MS has not been completely cured. Therefore, this review summarized the drugs investigated in the TMEV-induced disease (TMEV-IDD) model of MS, so as to provide medication guidance and theoretical basis for the treatment of virus-induced MS.

## Introduction

Theiler’s murine encephalomyelitis virus (TMEV) is a non-enveloped positive sense single-stranded RNA (ssRNA) Cardiovirus of the Picornaviridae family, which was found from some mice with flaccid paralysis of the hind legs among normal mice by Max Theiler in 1934 (Theiler, 1934; Pevear et al., 1987; Cusick et al., 2014). He collected a suspension of brain and spinal cord of these paralytic mice, intracerebrally injected into normal mice, and found that those injected mice showed the same symptoms as paralytic mice and these symptoms could be passed on to the next generation (Theiler, 1934). Viruses were isolated from the paralytic mice and named as Theiler’s murine encephalomyelitis viruses (TMEVs) (Theiler, 1934). Its RNA genome includes a 5′ untranslated region (UTR), an open reading frame (ORF), a 3’ UTR, and a poly A tail. Its ORF encodes a 2,303-amino acid polyprotein, which is post-translationally cleaved by viral proteases into L (leader protein), P1 (capsid region), P2 (midsection) and P3 (right portion) and finally into 12 mature proteins (Pevear et al., 1987; Tsunoda and Fujinami, 2010; Cusick et al., 2014). The four capsid proteins, VP1, VP2, VP3 and VP4, are structural proteins of TMEV and encoded by P1. The non-structural proteins including 2A, 2B, 2C, 3A, 3B, 3C, and 3D are encoded by P2 and P3, which are required for viral RNA replication (Oleszak et al., 2004; Tsunoda and Fujinami, 2010). 2A and 3C represent proteases, 2C is a nucleoside-triphosphatase, 3B is a binding site for internal ribosomal entry site (IRES), and 3D is a RNA-dependent RNA polymerase (Gerhauser et al., 2019).

TMEV is a rodent pathogen and induces mild gastroenteritis following oronasal infection while the infection of the central nervous system (CNS) is a rare event (Gerhauser et al., 2019). But experimental intracerebral infection of young adult mice causes flaccid paralysis of the hind limbs. Based on viral crystalline arrays in infected cells, ribonuclease T1 finger printing patterns, and neurovirulence following intracerebral inoculation of mice, TMEVs are divided into GDVII and TO groups, which are 90% identical at the nucleotide level and 95% identical at the amino acid level (Pevear et al., 1987; Tsunoda and Fujinami, 2010; Mecha et al., 2013; Li, 2016). The GDVII group is highly neurovirulent in mice, consists of GDVII and FA strains (Li, 2016). Infection intracerebrally with GDVII group into mice produces severe encephalitis resulting in death within one to two weeks (Yamada et al., 1991; Mecha et al., 2013; Li, 2016). TO subgroup is lowly neurovirulent in mice and includes DA (Daniels et al., 1981), WW (Stroop et al., 1981), TO4, BeAn8386, and Yale strains (Lorch et al., 1981). The intracerebral infection into mice with TO group induces a monophasic disease in resistant mouse strains such as C57BL/6, C57BL/10, C57/L and 129/J mice, which is characterized by a transient meningoencephalomyelitis and virus elimination (Li et al., 2015; Gerhauser et al., 2019). In contrast, highly susceptible SJL/J mice suffer from a biphasic disease consisting of an acute polioencephalomyelitis and a chronic demyelinating leukoencephalomyelitis with virus persistence (Lipton, 1975; Mecha et al., 2013; Li et al., 2015). Therefore, the course of Theiler’s murine encephalomyelitis (TME) depends on the virus and mouse strains as well as other factors including intestinal microbiota and stress (Gerhauser et al., 2019). However, TMEV is transmitted by the fecal-oral route in wild mice which do not result in clinical symptoms, which suggests the route of infection is important for the pathogenesis of the clinical symptoms. Susceptible mice infected intracerebrally by TMEV showed the infiltration of inflammatory cells such as T cells, B cells and microphages, gliosis, demyelination in the CNS, which is similar to the pathology of the human multiple sclerosis (MS) (Li et al., 2015).

MS is an inflammatory neurodegenerative disease in the CNS of human, which often occurs between the age of 20 to 40 (Libbey and Fujinami, 2021). The pathology of MS is characterized by the infiltration of the inflammatory cells, the astrogliosis, demyelination and axon damage in the CNS, which might be induced by genetic and environmental factors such as virus infection due to the etiology of MS is still unknown. The hallmarks of TME in SJL/J mice are similar to the MS, so the TMEV-induced disease (TMEV-IDD) is often used for exploring the pathogenesis and treatments of MS.

### Investigation of main drugs for MS in TMEV-IDD

At present, there are more than 20 drugs used to treat MS, including the marketed and clinical phase II and III drugs such as Interferon β-1a/b, Ocrelizumab, Dimethyl fumarate, Teriflunomide, Glatiramer acetate, Fingolimod, Opicinumab, CNM Au 8, Biotin, Elezanumab and so on. Based on different ways of these drugs to treat MS, they can be divided into three categories: immune modulators, Sphingosine-1-phosphate receptor (S1PR) regulators and nervous repair agents. However, the etiology of MS is still unknown, genetic, environmental, viral and autoimmune factors may induce MS, but not all drugs on the market are suitable for virus-induced MS. Thereby we summarized the drugs that have been investigated in TMEV-IDD model (Table 1).

**Table 1:** Drugs investigated in TMEV-IDD, EAE as well as MS patients.

Type	Name	Target	Model	References
Immune modulators	IFN-β 1a/b	Type I IFN pathway	TMEV	Li et al. (2015), Bühler et al. (2022), Bühler et al. (2023)
	Dimethylfumarate	IL-17, Nrf2	TMEV	Kobayashi et al. (2015)
	Natalizumab	α4 integrin, CD4^+^ T cells, CD8^+^ T cells	TMEV	Hirano et al. (2016)
	Teriflunomide	Microglia density, oligodendrocyte differentiation, glutamate	TMEV	Modica et al. (2017), Pol et al. (2019), Gilli et al. (2017)
	Glatiramer acetate	Foxp3、Il17a IL-4、IL-10 GA antibody	TMEV	Omura et al. (2018); Ure and Rodriguez (2002)
	Ocrelizumab	CD20	EAE	Häusler et al. (2018)
	Daclizumab	CD25	EAE	Rommer et al. (2020)
	Laquinimod	NK cells	EAE	Ott et al. (2019)
	Cladribine	T, B cells	EAE	Schroeter et al. (2022)
S1PR	Fingolimod	S1PR1	TMEV	Li et al. (2011)
	Siponimod	S1PR1, S1PR5	TMEV	Pol et al. (2023)
	Ozanimod	S1PR1, S1PR5	EAE	Musella et al. (2020)
	Ponesimod	S1PR1	EAE	Pouzol et al. (2019), Hou et al. (2021)
	Ceralifimod	S1PR1, S1PR5	MS	Dash et al. (2018)
	Amiselimod	S1PR1, S1PR4, S1PR5	MS	Kappos et al. (2018)
Nervous repair agents	Temelimab	Envelope protein	EAE	Curtin et al. (2015), Jakimovski et al. (2020)
	Opicinumab	LINGO-1	EAE	Ranger et al. (2018), Ruggieri et al. (2017)
	High-dose Biotin	Enhancing fatty acid and energy production	MS	Levy et al. (2022), Sedel et al. (2016), Rosko et al. (2019), Birnbaum and Stulc (2017)
	Elezanumab	Repulsive guidance molecule A	MS	Kalluri et al. (2023)
	CNM Au 8	Energetic metabolism	MS	Ren et al. (2023)

#### Immune modulators

Immune modulators are used in disease modification therapy (DMT) that is to control disease progression as the main goal and have been recommended for the long-term treatment of MS. These immune modulators interfere with immune responses in different ways. For example, IFN-β 1a/b is one of the longest-used drugs for MS, which exerts an antiviral effect mainly by regulating the inflammatory response through the Type I IFN pathway. The mRNA expression of IFN-β in TMEV-infected C57BL/6 was significantly higher than that in SJL/J mice, suggesting that IFN-β may be involved in the resistance against demyelination and virus clearance in C57BL/6 mice (Li et al., 2015; Bühler et al., 2022, 2023). However, IFN-β produced by neuroectodermal cells does not seem to play a critical role in the resistance of C57BL/6 mice against fatal and demyelinating disease (Li et al., 2015; Bühler et al., 2022, 2023). Dimethyl fumarate inhibited differentiation of T helper 17 (Th17) cells via suppressing NF-kB so that decreased the level of IL-17A mRNA, and enhanced the nuclear factor (erythroid-derived 2)-2 (Nrf2) antioxidant response in TMEV-IDD mice (Kobayashi et al., 2015). Natalizumab is an antibody against α4 integrin whose mRNA level was significantly up-regulated in TMEV-IDD mice. α4 integrin antagonist might inhibit the binding of α4 integrin to vascular cell adhesion molecule-1, thereby decreasing the numbers of TNF-α-producing CD4^+^ T cells and IFN-γ-producing CD8^+^ T cells in the CNS in the effector phase of TMEV-IDD (Hirano et al., 2016).Teriflunomide altered microglia density in corpus callosum and oligodendrocyte differentiation in basal ganglia, reduced possible excitotoxicity in the thalamus and basal ganglia by decreasing glutamate levels (Modica et al., 2017; Pol et al., 2019), so that it showed anti-inflammatory and antiviral properties, but there seemed to be no impact on disability progression and intrathecal antibody production in TMEV-IDD mice (Gilli et al., 2017).Glatiramer acetate neither enhanced viral loads nor suppressed antiviral immune responses, while it resulted in an increase in the Foxp3/Il17a ratio and IL-4/IL-10 production in TMEV-infected mice (Omura et al., 2018). Antibodies to Glatiramer acetate promote remyelination of spinal cord axons in TMEV-IDD (Ure and Rodriguez, 2002). In addition, Daclizumab, Laquinimod and Cladribine were not investigated in TMEV-IDD but in EAE model of MS. Daclizumab targets CD25 that is identical to the α subunit of the high-affinity IL-2 receptor, composed of the α, β, and γ subunits (Rommer et al., 2020). IL-2 cannot bind to the receptor after Daclizumab treatment, resulting in decreased T-cell activation and inflammation (Yang et al., 2010; Hao et al., 2011; Rommer et al., 2020). Laquinimod activates natural killer (NK) cells via the aryl hydrocarbon receptor, which augments their immunoregulatory functions in EAE by interacting with CD155^+^ dendritic cells (DC) (Ott et al., 2019). Noteworthy, the immunosuppressive effect of Laquinimod-activated NK cells was due to decreasing MHC class II antigen presentation by DC and not by increasing DC killing (Ott et al., 2019). Alemtuzumab and Metenkefalin/tridecactide is also used in DMT of MS, but its function is not showed in TMEV-IDD and EAE model. Based on above researches, it is suggested that IFN-β 1a/b, Dimethyl fumarate and Natalizumab may be effective in the treatment of virus-induced MS by regulating Type I IFN pathway, IL-17, CD4^+^ and CD8^+^ T cells, Teriflunomide may not play much of a role, and Glatiramer acetate may even be shown to play the opposite role. In addition, the roles and mechanisms of Daclizumab, Laquinimod and Metenkefalin/tridecactide in virus-induced MS remain to be investigated.

#### S1PR regulators

Sphingosine-1-phosphate (S1P) and S1P receptors (S1PR) are bioactive lipid molecules that are ubiquitously expressed in the human body (Bravo et al., 2022). S1P is the active terminal derivative of sphingosine metabolism, and act as a ligand for five different high-affinity cell surface receptors (S1PR1 to S1PR5) which belong to the superfamily of G protein-coupled receptors (GPCR) (Kihara et al., 2014; Bravo et al., 2022). The main S1P effect is regulating the lymphocyte egress from secondary lymphatic organs into the systemic circulation (Bravo et al., 2022). S1PR1 expressed on lymphocytes T and B is implicated in modulating the expression of some pro-inflammatory cytokines (Bravo et al., 2022). In CNS, S1P is released by cerebral sphingosines and its receptor is expressed by all types of brain cells, including neurons, astrocytes, and oligodendrocytes (Matloubian et al., 2004; Oo et al., 2007). The discovery of the S1P/S1PR complex in the CNS was used as a treatment for MS by the modulation of the signaling of this complex. Therefore, S1PR regulators can prevent lymphocytes from leaving the lymph node to reach the site of inflammation, thereby reducing damage to the nervous system and decreasing the recurrence of MS, including Fingolimod, Siponimod, Ozanimod, Ponesimod, Ceralifimod, Amiselimod.

Bioactivity of Fingolimod was confirmed by reduced numbers of mononuclear cells in the spleen and blood in TMEV-IDD mice, but it had no effect on disability progression, viral load, and serum antibody response although it can cross the blood brain barriers (Li et al., 2011). Siponimod suppressed ventricular enlargement, suggesting reduced TMEV-induced inflammation in lateral ventricle, but it did not demonstrate a significant impact on neurodegeneration, spinal volume, or lesion volume in the TMEV mouse model (Pol et al., 2023). Data of Ozanimod, Ponesimod, Ceralifimod and Amiselimod investigated in TEMV-IDD model is still pore.

#### Nervous repair agents

Different from the immune modulators and S1PR regulators, nervous repair agents mainly promote remyelination after the CNS injury by regulating the generation of myelin, such as Opicinumab, CNM Au 8, Biotin, Temelimab, RNS 60, Elezanumab and so on. Currently, no evidence was found in the TMEV model to show the mechanism of these drugs treating MS, only Temelimab and Opicinumab were investigated in EAE model of MS. Temelimab is humanized IgG4 monoclonal antibody targeting the envelope (Env) protein of an active element from the human endogenous retrovirus type W family, also named multiple sclerosis associated retrovirus (MSRV) (Curtin et al., 2015; Jakimovski et al., 2020). Temelimab can prevent exacerbations of MSRV-Env induced EAE (Curtin et al., 2015). Opicinumab is antibody against leucine-rich repeat and immunoglobulin-like domain-containing nogo receptor-interacting protein 1(LINGO-1) which negatively regulates the differentiation of oligodendrocytes to promote remyelination but does not affect immune function in EAE model (Ruggieri et al., 2017; Ranger et al., 2018).

In addition to these two drugs, other nervous repair agents are still in the clinical trial stage such as Biotin, Elezanumab and CNM-Au8. Biotin is a water-soluble vitamin B-complex and an essential cofactor for five carboxylases (Levy et al., 2022), which attenuates oxidative stress, mitochondrial dysfunction, lipid metabolism alteration and 7β-hydroxycholesterol-induced cell death in 158 N murine oligodendrocytes (Sedel et al., 2016), and might promote myelin synthesis by enhancing fatty acid production and increasing energy production by acting as a coenzyme in oligodendrocytes and neurons (Rosko et al., 2019; Levy et al., 2022). Thereby high-dose biotin has been used to treat MS, it was safe and well tolerated, but of no demonstrable long-term benefit for MS (Birnbaum and Stulc, 2017). Elezanumab is a fully human monoclonal antibody directed against repulsive guidance molecule A that is a potent modulator of axonal growth, myelination, and downstream immunoregulatory molecules (Kalluri et al., 2023). CNM-Au8 is a suspension of faceted, clean-surfaced gold nanocrystals that catalytically improves energetic metabolism in CNS cells, supporting neuroprotection and remyelination as demonstrated in multiple independent preclinical models (Ren et al., 2023).

### Investigation of gut microbiota in TMEV-IDD as adjunctive drugs for MS

Microorganisms are closely related to human life and can be seen everywhere in daily life. Many scholars have found that intestinal microbes participated into the development of MS through the gut-brain axis. The intracerebral inoculation of TMEV in SJL/J mice neither decreased microbial diversity nor changed overall microbiome patterns, while it leaded to a moderate dysbiosis of intestinal microbiota (Carrillo-Salinas et al., 2017; Omura et al., 2020).

TMEV infection in SJL/J mice increased abundance of individual bacterial genera Marvinbryantia and Coprococcus which showed the strong correlation with CNS transcriptome: Marvinbryantia with eight T-cell receptor (TCR) genes and with seven immunoglobulin (Ig) genes, and Coprococcus with gene expressions of not only TCRs and IgG/IgA, but also major histocompatibility complex (MHC) and complements (Omura et al., 2020). After the oral administration of antibiotics (ABX) to TMEV-infected SJL/J mice, it was observed that the abundance of Bacteroidetes and Firmicutes was decreased and the abundance of Actinobacteria and Proteobacteria phyla was increased (Mestre et al., 2019). ABX treatment prevented motor dysfunction and limited axon damage in TMEV-infected SJL/J mice, which modified neuroimmune responses to TMEV dampening brain CD4^+^ and CD8^+^ T infiltration during the acute phase (Carrillo-Salinas et al., 2017; Mestre et al., 2019). Furthermore, ABX treated mice displayed lower levels of CD4^+^ and CD8^+^T cells in cervical and mesenteric lymph nodes and limited IL-17 production *ex vivo* (Carrillo-Salinas et al., 2017; Mestre et al., 2019). Increased mortality to TMEV was observed after ABX cessation at 28dpi (Carrillo-Salinas et al., 2017). On the chronic phase, mice that survived after ABX withdrawal and recovered microbiota diversity showed subtle changes in brain cell infiltrates, microglia and gene expression of cytokines (Carrillo-Salinas et al., 2017). Accordingly, the surviving mice of the group ABX-TMEV displayed similar disease severity to TMEV mice (Carrillo-Salinas et al., 2017), while the gut microbiota recolonization induced worsened motor function and axonal integrity after ABX treatment (Mestre et al., 2019). Therefore, it was evidenced in TMEV-IDD model that the regulation to abundance of some gut microbiota by ABX might improve immunopathology of MS and facilitate to treatment of virus-induced MS.

However, abundance of gut microbiota is regulated not only by ABX but also probiotics. A variety of studies have suggested that probiotics would exert benefits when using in combination with current MS therapy as oral nontoxic immunomodulatory agents such as Vivomixx (Mestre et al., 2020). Vivomixx is a multi-species probiotic composed of *Lactobacillus paracasei* DSM 24734, *Lactobacillus plantarum* DSM 24730, *Lactobacillus acidophilus* DSM 24735, *Lactobacillus delbruckeii subspecies bulgaricus* DSM 24734, *Bifidobacterium longum* DSM 24736, *Bifidobacterium infantis* DSM 24737, *Bifidobacterium breve* 24,732 and *Streptococcus thermophilus* DSM 24731 (Mestre et al., 2020). Vivomixx administration in TMEV-infected mice improved the motor disability of mice and reduced microgliosis, astrogliosis and leukocyte infiltration in CNS through the increased abundance of many taxa such as Bacteroidetes, Actinobacteria, Tenericutes and TM7 (Mestre et al., 2020).

Normally, abundance of “good bacteria” such as *Bifidobacterium* have been believed to be beneficial for diseases, while “bad bacteria” such as pathogenic *Helicobacter pylori* are assumed to be always detrimental for hosts. However, *H. pylori* and another bad bacterium *Clostridium perfringens* type A have been proposed to be protective against MS in TMEV animal model (Park et al., 2017; Tsunoda, 2017). *H. pylori* evaded the host immune response by inducing Th2 cells and regulatory T cells (Tregs) that produce anti-inflammatory interleukin (IL)-10 (Park et al., 2017). Tregs suppressed pro-inflammatory Th1/Th17 responses to *H. pylori* and the induction of encephalitogenic Th1/Th17 cells, which was proposed to be protective against MS (Park et al., 2017). In a word, gut microbiota is involved in the development of TMEV-IDD through immunopathology, suggesting that improving the dysbiosis of gut microbiota with probiotics facilitates to the treatment of virus-induced MS. So far, modulation of gut microbiota has mostly used as an adjunctive therapy for MS.

To sum up, IFN-β 1a/b, Dimethyl fumarate and Natalizumab may be effective in the treatment of virus-induced MS by regulating Type I IFN pathway, IL-17, CD4^+^ and CD8^+^ T cells. Teriflunomide, Fingolimod and Siponimod may not play much of a role, and Glatiramer acetate may even be shown to play the opposite role. The mechanisms and efficacy of immune modulators (Daclizumab, Laquinimod, Metenkefalin/tridecactide), S1PR regulators (Ozanimod, Ponesimod, Ceralifimod and Amiselimod) and nervous repair agents (Opicinumab, CNM Au 8, Biotin, Temelimab, RNS 60, Elezanumab) in the treatment of virus-induced MS remain to be investigated. In addition, Daclizumab, Laquinimod and Temelimab may be helpful in treating immune-induced MS based on their studies in EAE models of MS. Furthermore, modulation of gut microbiota by probiotics or antibiotics may facilitate to treat virus-induced MS as an adjunctive therapy for MS.

## Author contributions

LL: Conceptualization, Funding acquisition, Project administration, Supervision, Writing – original draft, Writing – review & editing, Methodology. RZ: Writing – review & editing. LS: Supervision, Writing – review & editing.
